# Improving pediatric post-acute kidney injury care: considerations for the clinician

**DOI:** 10.1080/0886022X.2026.2672176

**Published:** 2026-05-20

**Authors:** Tal Golan Lagziel, Aseel Al-Dmour, Rahul Chanchlani, Cal H. Robinson

**Affiliations:** ^a^ Department of Paediatrics, Division of Nephrology, The Hospital for Sick Children, Toronto, Canada; ^b^ Department of Pediatrics, Division of Nephrology, McMaster Children’s Hospital, Hamilton, Canada; ^c^ Department of Health Research Methods, Evidence, and Impact, McMaster University, Hamilton, Canada; ^d^ Child Health Evaluative Sciences, Research Institute, The Hospital for Sick Children, Toronto, Canada; ^e^ Institute of Health Policy, Management and Evaluation, University of Toronto, Toronto, Canada

**Keywords:** Acute kidney injury, pediatric, child, chronic kidney disease, hypertension, follow-up

## Abstract

Acute kidney injury (AKI) is a frequent complication among hospitalized children, particularly during critical illness, and is associated with higher mortality rates and prolonged hospitalization. Accumulating evidence indicates that the consequences of pediatric AKI extend beyond the acute hospitalization and have strong associations with subsequent adverse kidney outcomes and hypertension. A review of the pediatric literature reveals a paucity of data defining the optimal timing, frequency, and components of post-AKI follow-up in children to mitigate long-term morbidity. In this review, we will address the potential sequelae of AKI among hospitalized children, including the temporal spectrum of acute kidney disease and chronic kidney disease. We will further discuss risk factors associated with adverse long-term outcomes and recommended follow-up surveillance after childhood AKI. In addition, we will review emerging biomarkers that may help identify children at higher risk for adverse outcomes following AKI.

## Childhood acute kidney injury

### AKI incidence

Acute kidney injury (AKI) is a common condition among hospitalized children, with variable incidence by age, underlying medical complexity, the hospitalization setting (intensive care units [ICU] vs. general pediatric wards), illness severity, and global regions (Table 1) [1–3,7]. The Assessment of Worldwide Acute Kidney Injury, Renal Angina and Epidemiology in critically ill children (AWARE) study, demonstrated additional variability in AKI incidence among 4683 ICU patients, with daily prevalence rising from 15% on the first day ICU admission to over 20% by the seventh day [2]. Notably, most AKI cases developed within the first four days of ICU admission. AKI may be further classified into community-acquired or hospital-acquired, each with distinct etiological patterns, incidence, and prognosis [8]. Distinct etiological patterns are also observed when comparing childhood AKI across high-, upper-middle-, lower-middle-, and low-income countries [9]. In high-income countries, AKI was more frequently associated with hypotension, postoperative complications, and dehydration, whereas in lower-middle- and low-income countries, AKI was more commonly related to infections, nephrotoxic medications, and primary kidney diseases.

**Table 1.** Acute kidney injury incidence in different hospitalized pediatric populations.

Pediatric population	AKI incidence	Study description
Hospitalized children, aged 1 mo-18yo	26%	Meta-analysis of 94 studies, with 202,694 participants from 26 countries [1]
Critically-ill children and young adults, aged 3 mo-25yo	27%	Prospective-observational study, in 32 pediatric intensive care units [2]
Not critically-ill hospitalized children, aged 1 mo-18yo	9%	Single tertiary center, prospective-observational study [3]
Infants and children post-cardiac surgery	9%	Retrospective single center study, in 150 infants and children underwent cardiac surgery [4]
	42%	Prospective observational multicenter cohort study [5]
Hospitalized pediatric oncology patients	11%	Retrospective multi-center study, with 9828 Chinese participants [6]
Neonates admitted to intensive care units	30%	Analysis of the AWAKEN study; medical records of neonates admitted to 24 NICUs were reviewed [7]

AKI: acute kidney injury, AWAKEN: Assessment of Worldwide Acute Kidney Injury Epidemiology in Neonates.

### AKI definitions

AKI is defined as a rapid decline in renal function, reflected by an increase in serum creatinine and/or a decrease in urine output. The rise in creatinine must occur within a defined short-term timeframe, while the reduction in urine output must persist for a specified minimum duration [10]. In 2012, Kidney Disease: Improving Global Outcomes (KDIGO) published clinical practice guidelines for AKI, which are currently in the process of being updated [10]. These KDIGO guidelines define AKI as a rise in serum creatinine of ≥0.3 mg/dL (≥26 µmol/L) within 48-h; an increase of ≥50% from baseline creatinine within seven days; or urine output of <0.5 mL/kg/hour for more than six hours [11]. Modified AKI definitions have been proposed for neonatal AKI, including a urine output threshold of ≤1 mL/kg/hour over a 24-h period between days 2–7 after birth or a serum creatinine increase of ≥50% within seven days from the previous lowest value [12]. The modifications of the KDIGO-based AKI definition in neonates, particularly regarding the reference creatinine value (‘baseline’ vs. ‘previous lowest’), reflects the unique physiology of early life. During the first days after birth, neonatal serum creatinine is influenced by maternal creatinine levels and immature GFR, among other factors [13]. Over the subsequent weeks, serum creatinine typically declines, rendering the concept of a fixed ‘baseline’ inappropriate. Consequently, neonatal AKI definitions rely on comparison with the lowest previously recorded creatinine value rather than a static baseline [7].

It can be observed that current AKI definitions rely on markers of kidney function (serum creatinine and urine output), not injury. In response to volume depletion, functional markers change appropriately and may not reflect true tissue injury. New AKI classifications, based on the combination of functional and injury biomarkers (e.g., urinary neutrophil gelatinase–associated lipocalin [NGAL]), have been proposed to differentiate functional AKI (functional biomarker positive, injury biomarker negative), subclinical AKI (functional biomarker negative, injury biomarker positive), and damage-associated AKI (functional and injury biomarker positive) [14].

### AKI etiology

Etiologically, AKI may result from functional causes that impair renal perfusion, post-kidney obstruction of urinary outflow, or structural causes involving tissue injury at the level of the glomeruli, tubules, vasculature, interstitium, or a combination thereof [15]. For example, iodinated contrast media (ICM)–induced AKI is thought to result from a combination of direct tubular epithelial cell toxicity and endothelial dysfunction at the level of the peritubular and glomerular capillaries, ultimately leading to tubular necrosis [16]. Different AKI etiologies are associated with distinct pathophysiological mechanisms and therapeutic approaches, supporting the concept of AKI as a syndrome [17].

Various epidemiologic studies in hospitalized children have reported differing patterns of AKI specific etiology. In a US study, the most frequent causes of AKI among hospitalized children were renal ischemia and nephrotoxic medication exposures, whereas primary renal diseases and hemolytic uremic syndrome (HUS) were less common [18]. Conversely, a UK study found that nearly half of AKI cases were secondary to HUS, and surgical interventions for congenital heart disease accounted for 60% of neonatal AKI cases [19]. The causes of childhood AKI also vary globally, with higher incidence of community-acquired AKI due to infection, dehydration, and HUS in low-to-middle-income countries [9,20]. It is well recognized that certain pediatric populations are at increased risk of developing AKI, owing to disease-specific factors (e.g., congenital heart disease, cancer, stem cell or solid organ transplant recipients, diabetes, sickle cell disease, underlying kidney disease), state-specific factors (e.g., critical illness, trauma, dehydration, hypotension), and procedure-specific factors (e.g., major surgery, nephrotoxic medications, iodine-based radiocontrast) [21]. It is worth noting that the risk of developing AKI is increased in the presence of multiple contributing factors, such as exposure to ICM in patients with preexisting renal impairment or concomitant treatment with other nephrotoxic agents [10].

### Long-term kidney outcomes of AKI

The short term complications of AKI have been comprehensively reviewed elsewhere and include electrolyte and acid-base disturbances, prolonged hospitalization, prolonged mechanical ventilation, hypertension, and increased mortality [21–23]. To mitigate the risk of further deterioration in kidney function, initial management of AKI should focus on avoidance of nephrotoxic agents, adjustment of medication dosing according to current GFR, management of electrolyte abnormalities with supplementation or pharmacologic binders, and optimization of blood pressure and fluid balance; in case of oligoanuric AKI, not in the setting of kidney hypoperfusion, fluid intake should be restricted to the sum of insensible losses and measured urine output [24].

As the primary focus of this review, the long-term complications of AKI have redefined its perception. AKI is no longer viewed as a transient and fully reversible syndrome. Children who survive an episode of dialysis-treated AKI are at increased risk of developing chronic kidney disease (CKD), hypertension, and recurrent AKI [25]. The risk of CKD is highest during the first year post-AKI and remains elevated for >5-years, suggesting a window of opportunity for early detection and intervention [25]. A recent meta-analysis of 38 studies including 14,892 hospitalized children with AKI reported that the cumulative incidence of CKD was 16%, mortality was 6%, proteinuria was 19%, and hypertension was 13% [26]. Among 24 studies including non-AKI controls, children that developed AKI were found to be at 57% higher odds of developing CKD and 84% higher odds of mortality. Among 4173 children that survived an episode of AKI not treated with acute kidney replacement therapy, 18% developed major adverse kidney events (MAKE; death, kidney failure, or CKD) over median 10-year follow-up [27]. Childhood AKI survivors were at 2–4 times higher risk of MAKE, hypertension, and subsequent AKI than propensity score-matched hospitalized controls without AKI.

### Non-kidney sequelae of childhood AKI

Childhood AKI been associated with long-term sequelae beyond the kidney. Up to 20% of children that experience AKI develop hypertension over long-term follow-up, which is an established risk factor for adult cardiovascular disease and mortality [25,27,28]. Although studies evaluating the long-term cardiovascular outcomes of pediatric AKI are lacking, AKI in adults is strongly associated with an increased risk of cardiovascular mortality, heart failure, acute myocardial infarction, and stroke [29]. Recurrent and severe pediatric AKI (neonatal and children AKI) has been independently associated with worse neurodevelopmental and cognitive outcomes [30–32]. Development of CKD post-AKI may further increase the risk of poor neurocognitive outcomes [33]. Studies of pediatric ICU and sepsis survivors demonstrate that severe AKI is associated with higher rates of functional disability and worse health-related quality-of-life post-discharge [30,34–36]. Childhood AKI has also been found to adversely impact growth. A meta-analysis of 17 studies including 3586 children reported that those with AKI had significantly lower length (mean difference −0.37) and weight z-scores (mean difference −0.29) over 1–11 year follow-up [37]. AKI also has deleterious effects on immune system function and is associated with an increased risk of infections [38–42]. Although the risk of infection is highest in the first month post-AKI, emerging data suggests that these effects may be prolonged, with evidence of higher infection risk up to 1-year after AKI [41,42]. The non-kidney sequelae of childhood AKI are likely under-recognized and under-reported, but should be considered by clinicians involved in post-AKI care.

## The continuum of AKI, AKD, and CKD

Current AKI definitions restrict the duration of AKI to 1-week from injury onset [43]. CKD is characterized by structural or functional kidney abnormalities that are present for a minimum duration of three months, including decreased GFR (<90 mL/min/1.73m^2^); albuminuria (urine albumin:creatinine ratio >3mg/mmol); abnormal pathology, imaging, or other laboratory markers (e.g., electrolytes and acid-base abnormalities related to tubular disorders) [44]. Increasingly, the timing and extent of AKI recovery is recognized as key prognostic factor for subsequent kidney outcomes [45,46]. This has led to the concept of acute kidney disease (AKD). AKD is defined as kidney damage persisting for <3 months, with AKI constituting a subset of this spectrum [47,48]. For simplicity, AKI is considered part of the AKD spectrum; when an increase in serum creatinine persists for more than 7 days but less than 3 months, the condition is classified as AKD.

**Figure 1.**
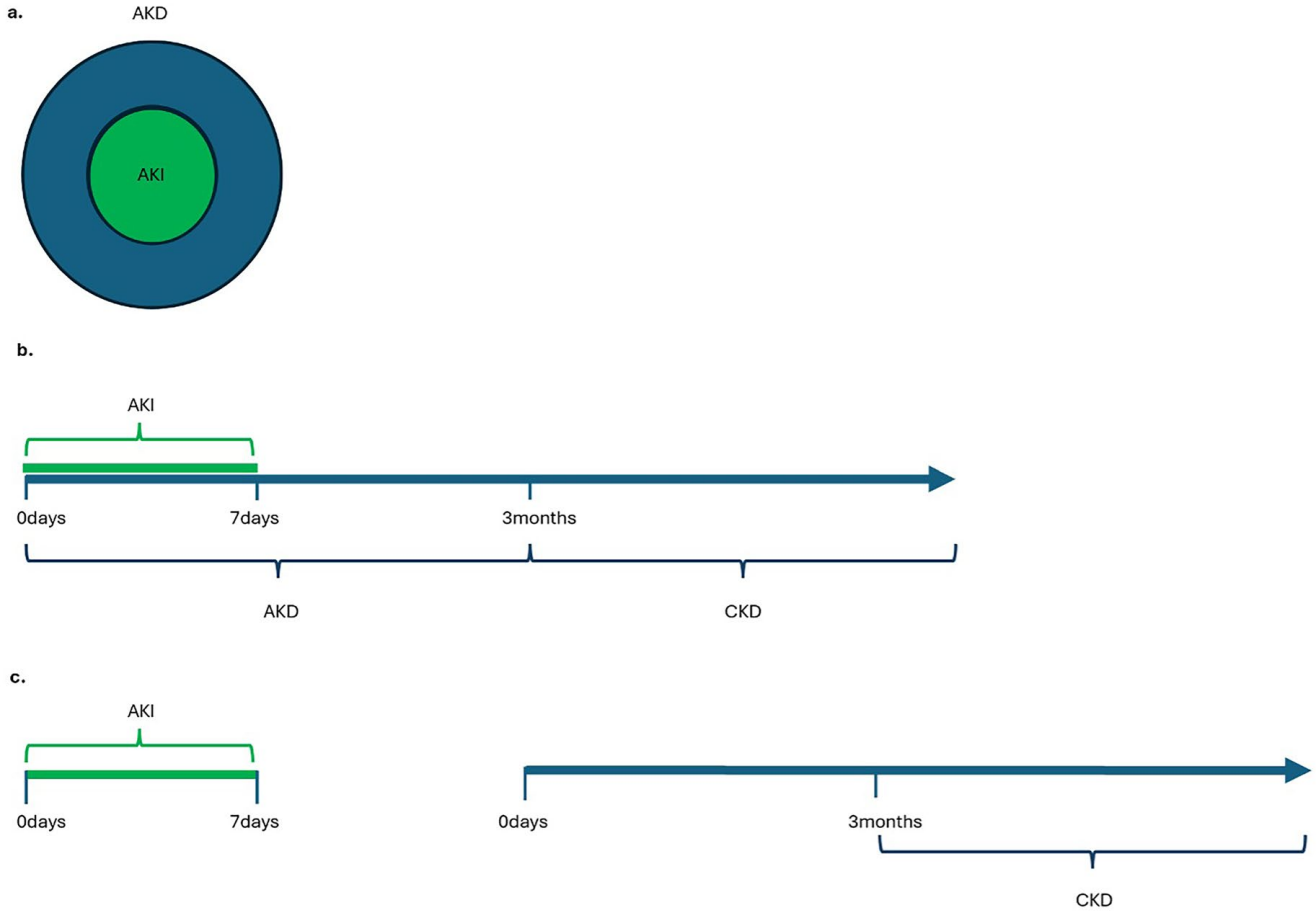
Temporal relationships of acute kidney injury, acute kidney disease, and chronic kidney disease. AKI: acute kidney injury, AKD: acute kidney disease, CKD: chronic kidney disease Figure 1a illustrates that AKI represents a subset of AKD, with the persistence of elevated serum creatinine serving as the defining criterion for non-AKI AKD, as highlighted in Figure 1b. Figure 1b demonstrates the temporal continuum of AKI as a sentinel event that may lead to sustained kidney dysfunction, classified as AKD within the first 3-months and CKD if it persists beyond 3-months. Figure 1c demonstrates complete AKI resolution within 7-days without subsequent AKD, followed by development of CKD at a future timepoint. The future onset of kidney dysfunction starts at ‘0 days’ and persists for 3-months, at which point it is classified as CKD.

AKI, AKD, and CKD represent a temporal continuum, whereby patients with AKI are at increased risk of developing AKD, and those with AKD are at increased risk of progressing to CKD [49–53]. However, children with AKI that have complete recovery without AKD are still at increased risk of CKD. Among 528 hospitalized children with AKI, 57% progressed to AKD [53]. Among children with AKD, 46% progressed to CKD, compared to 19% of children without AKD. Figure 1 illustrates the suggested temporal relationship between AKI, AKD, and CKD. The early post-AKI period may be a critical window of opportunity for intervention to promote kidney recovery and prevent irreversible progression.

### CKD risk factors after childhood AKI

Beyond AKD, several patient- and disease-related risk factors for progression from AKI to CKD have been identified (Table 2). Evidence has shown a graded correlation between increasing AKI severity and subsequent risk of CKD progression [54,55]. CKD risk also depends on AKI etiology. Childhood AKI due to nephrotoxic medication exposure, ischemic acute tubular necrosis, HUS, and snake envenomation, conditions characterized by profound structural kidney damage and irreversible fibrotic changes, are associated with increased risks of subsequent CKD [56–59]. Fibrosis, driven by the generation of myofibroblasts and extracellular matrix deposition throughout the renal compartments (glomeruli, tubulointerstitium, and vasculature), represents the principal molecular mechanism underlying the transition from AKI to CKD [60]. Moreover, patient-related factors including prematurity and low birth weight (among neonates), congenital heart disease, cardiac surgery, malignancy treated with chemotherapy, stem cell and solid organ transplantation are independent risk factors for CKD that further increase the risk of CKD following episodes of AKI [21,61,62].

**Table 2.** Risk factors for chronic kidney disease after pediatric acute kidney injury.

Patient-related risk factors	AKI-related risk factors
Neonates, especially extremely preterm and very low birthweight	Acute kidney disease, prolonged AKI duration, and functional non-recovery
Cancer and chemotherapy treatment	AKI severity and acute kidney replacement therapy receipt
Stem cell and solid organ transplant recipients	Recurrent AKI
Congenital heart disease and cardiac surgery	AKI due to primary kidney disease (immune-mediated glomerular disease, tubulointerstitial disease, hemolytic uremic syndrome, or ischemic acute tubular necrosis)
Diabetes and diabetic ketoacidosis	AKI due to lower urinary tract obstruction (e.g., posterior urethral valves)
Sickle cell disease	AKI secondary to nephrotoxic medications or snake envenomation
Chronic liver disease	AKI during critical illness and need for mechanical ventilation

AKI: acute kidney injury.

### Neonatal AKI outcomes

Neonatal AKI, defined as AKI occurring within the first month of life, is a common complication among neonates admitted to the ICU and represents a significant risk factor for both acute morbidity and mortality and long-term sequelae, including progression to CKD, as well as hypertension and proteinuria [7,25, 27,63,64]. Prematurity, which associated with reduced nephron endowment due to interrupted nephrogenesis, and low birth weight are risk factors for CKD [63,65]. Data on CKD incidence following neonatal AKI are limited due to the prolonged follow-up period needed to assess long-term outcomes and heterogenous definitions of neonatal AKI and CKD used among studies [66].

## Stratifying CKD risk using novel biomarkers and artificial intelligence

A proposed shift in terminology from AKI to ‘kidney attack’ mirrors the concept ‘heart attack’, with creatinine-elevated AKI likened to ST-segment elevation myocardial infraction (STEMI) and the possibility of non-creatinine-elevated AKI to non-ST-segment elevation myocardial infraction (NSTEMI), underscoring the need for specific kidney tissue injury biomarkers analogous to troponin as a marker for myocardial injury [67]. However, most AKI biomarker research had focused on identifying biomarkers for early AKI detection (e.g., NGAL, kidney injury molecule [KIM]-1) [68–72] or differentiating functional vs. structural injury mechanisms (e.g., urine calprotectin:creatinine ratio) [73].

Despite the expanding potential of novel biomarkers for early AKI detection, AKI subtype differentiation, and prediction of progression among children with preexisting CKD [74], their utility for evaluating CKD risk after childhood AKI remains uncertain (Table 3). Biomarkers that are effective for early AKI detection have been shown to have limited predictive ability for subsequent CKD. Cooper et al. evaluated 33 pediatric patients who developed AKI following cardiopulmonary bypass surgery for congenital heart disease [75]. Although children that developed AKI exhibited higher levels of well-studied urinary biomarkers, including interleukin-18 (IL18) and liver-type fatty acid–binding protein (L-FABP), compared with controls who did not develop postoperative AKI, these did not correlate with subsequent CKD occurrence. Greenberg et al. conducted a 5-year follow-up of 49 patients who developed AKI after undergoing cardiopulmonary bypass surgery and found that none of the urinary biomarkers studied (IL18, KIM-1, monocyte chemoattractant protein-1, chitinase-3-like protein 1 [YKL-40], or NGAL) were associated with subsequent CKD [76].

**Table 3.** Emerging biomarkers for early diagnosis of acute kidney injury, the risk of subsequent chronic kidney disease, and chronic kidney disease progression.

Biomarker	Biological source	Physiological mechanism	AKI diagnosis	AKI recovery	CKD progression
Neutrophil gelatinase-associated lipocalin	Serum, urine	Tubular epithelial injury	+	+	+/-
Kidney injury molecule 1	Serum, urine	Tubular epithelial injury; facilitates phagocytosis of damaged cells.	+	+	+/-
Tissue inhibitor of metalloproteinases-2 and insulin-like growth factor-binding protein 7	Urine	Tubular cell cycle arrest marker	+	+/-	+/-
Soluble tumor necrosis factor receptor 1 and 2	Serum	Response to kidney function loss and inflammatory marker	–	+	+
Interleukin 18	Urine	Proximal tubular pro-inflammatory cytokine; could identify AKI with inflammatory component as therapeutic target	+	–	–
Monocyte chemoattractant protein-1	Urine	Tubular epithelial injury, particularly ischemic	+	–	–
Calprotectin: Creatinine ratio	Urine	Inflammatory marker (released from activated neutrophils)	+	–	–
Liver-type fatty acid-binding protein	Urine	Antioxidant properties that may prevent AKI progression	+	–	–

AKI: acute kidney injury, CKD: chronic kidney disease; IGFBP7: insulin-like growth factor-binding protein 7; IL18: interleukin 18; KIM-1: kidney injury molecule 1; L-FABP: liver-type fatty acid-binding protein; MCP-1: monocyte chemoattractant protein-1; NGAL: neutrophil gelatinase–associated lipocalin; sTNFR1 and 2: soluble tumor necrosis factor receptor 1 and 2; TIMP-2: tissue inhibitor of metalloproteinases-2. + indicates a potential role in clinical practice based on available data, ± indicates conflicting evidence, and - indicates that the biomarker is not useful or has not been studies for this purpose.

Further research in pediatric AKI populations with available long-term follow-up data is needed to identify biomarkers capable of predicting CKD occurrence and progression. A promising direction is to focus on molecular pathways implicated in the AKI-to-CKD transition, particularly maladaptive kidney repair characterized by persistent inflammation, myofibroblast activation, and related processes [60].

As in other medical subspecialties, the integration of artificial intelligence (AI) into pediatric nephrology has become increasingly prevalent. Hu and Raina reviewed the potential applications of AI in this field, including AKI prediction models, early detection of AKI, identification of AKI subphenotypes, early recognition of the need for kidney replacement therapy (KRT), and support for clinicians in the management of KRT [77]. Notably, AI-based tools have demonstrated the ability to identify adults with CKD at risk for progression [78] and to predict adverse outcomes of AKI, including the risk of progression to CKD following an AKI episode. Nateghi Haredasht et al. demonstrated that the use of machine learning-based prediction models in adults who developed stage 3 AKI during ICU admission effectively predicts the development of CKD at 3 and 6 months following the AKI episode, as well as mortality. They further proposed implementing the algorithm upon ICU discharge to identify patients at higher risk of progression to CKD, a recognition that will guide follow-up decision-making [79]. The capacity of AI to dynamically capture data throughout the hospitalization of patients with AKI, together with its ability to integrate large-scale datasets [80], renders it a potentially valuable tool for predicting long-term outcomes following AKI, including transition to CKD.

## Long-term follow up, timing and duration of post-pediatric AKI surveillance

Based upon the accumulating evidence of long-term complications, children who survive an AKI episode require systematic follow-up (Table 4). The goals of post-AKI surveillance are to promote kidney functional recovery, reduce the risk of AKI recurrence, avoid future nephrotoxic injury, and detect CKD, hypertension, and other AKI sequelae early to provide opportunities for intervention to prevent long-term kidney failure and cardiovascular events [21,81,82]. However, despite the theoretical rationale, post-discharge monitoring and follow-up guidelines remain insufficient. Canadian studies have found that only half of pediatric ICU-AKI survivors have serum creatinine re-measured and less than 20% of pediatric ICU or dialysis-treated AKI survivors are seen by a nephrologist within 1-year of discharge [83–85].

**Table 4.** Proposed standardized post-AKI follow-up.

AKI stage	Preexisting CKD	CKD risk factors*	Timing of initial follow-up	Follow-up provider	Surveillance components
Upon discharge: Unresolved AKI and AKD					Assess kidney health Review of AKI history BP measurement eGFR measurement Proteinuria measurement Patient and family education Discuss AKI diagnosis Strategies to improve long-term kidney health (avoid dehydration, nephrotoxins, sick day rules) Medication review, and eGFR dosing adjustment if needed Additional evaluation, on case-by-case basis: Additional labs (serum and urine) Kidney ultrasound 24 hr ambulatory blood pressure monitoring
Any	–	–	Up to 1 month	Nephrologist	
Upon discharge: Resolved AKI					
Stage 3 ± KRT	CKD3+	–	1 month	Nephrologist	
Stage 2 or recurrent	CKD2	–	1–3 months	Nephrologist or PCP	
Stage 1	No	Yes	3–6 months	Nephrologist or PCP	
Stage 1	No	No	6–12 months	PCP	

AKI: acute kidney injury, AKD: acute kidney disease, CKD: chronic kidney disease, KRT: kidney replacement therapy, PCP: primary care provider eGFR: estimated glomerular filtration rate, CKD3+: chronic kidney disease stage ≥3 (eGFR <60mL/min/1.73m^2^), CKD2: chronic kidney disease stage 2 (eGFR 60–89mL/min/1.73m^2^), *CKD risk factors are listed in Table 2.

**Figure 2.**
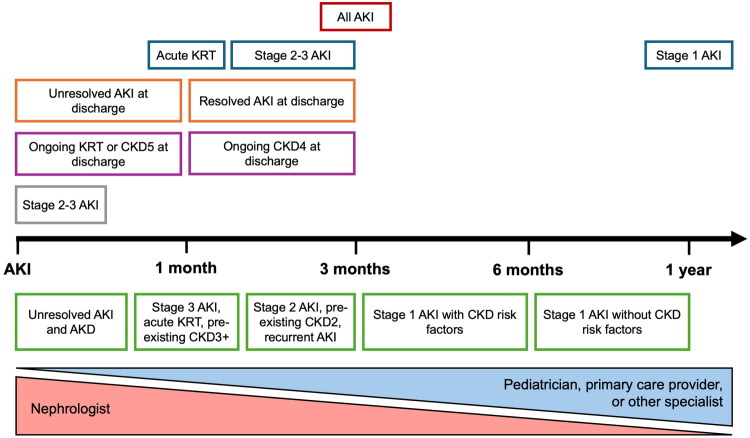
Variability of existing recommendations for initial pediatric AKI follow-up and suggested standardized follow-up. Figure 2 illustrates the variability between existing recommendations for initial post-AKI follow-up timing. The timeline (in black) indicates time post-AKI discharge. Text boxes above the timeline represent existing institutional, national, and international recommendations for initial post-AKI follow-up timing (red: Kidney Diseases Improving Global Outcomes [KDIGO] and pediatric Acute Disease Quality Initiative [ADQI] [18,40], blue: AlNafisi et al. [2025] [83], orange: Children’s Mercy Hospital [Kansas City, Missouri] [91], purple: UK Renal Association [2019] [92], and grey: Cincinnati Children’s Hospital [Cincinnati, Ohio] [93]. Text boxes in green below the timeline are suggested standardized post-AKI initial follow-up visit timings, based on current evidence to risk stratify pediatric AKI survivors. The lower panel depicts recommended follow-up provider based on AKI severity, resolution, preexisting CKD status, and CKD risk factors (listed in Table 2). AKI: acute kidney injury, KRT: kidney replacement therapy, CKD5: chronic kidney disease stage 5 (eGFR <15mL/min/1.73m^2^), CKD4: chronic kidney disease stage 4 (eGFR 15–29mL/min/1.73m^2^), CKD3+: chronic kidney disease stage ≥3 (eGFR <60mL/min/1.73m^2^), CKD2: chronic kidney disease stage 2 (eGFR 60–89mL/min/1.73m^2^), AKD: acute kidney disease

Currently, standardized post-AKI follow-up guidelines for children are lacking, and considerable variability exists in clinical practice [86]. Figure 2 illustrates the variability between existing post-AKI surveillance recommendations, as well as suggested follow-up timeframes based on current evidence. However, considerable uncertainty remains regarding whether transient stage 1 AKI requires follow-up, which providers should oversee follow-up (i.e., pediatric nephrologists, pediatricians, or primary care providers), which parameters should be monitored (blood pressure, growth, kidney function, electrolytes, urinalysis, proteinuria quantification), when follow-up assessments should be timed, how long should follow-up be continued, and how follow-up should be tailored according to CKD risk factors and AKI history.

### Timing and duration of post-AKI surveillance

The initial post-AKI follow-up visit should occur within 1–12 months after AKI, with timing dependent on AKI severity, duration, extent of renal recovery by discharge, presence of preexisting CKD, and CKD risk factors (Table 4 and Figure 2) [21,81]. The first 1–2 years after AKI occurrence represent the highest risk period for CKD, hypertension, or recurrent AKI events, underscoring the need for close surveillance during this interval [25,27]. The frequency and duration of subsequent follow-up after the initial visit should be individualized based on kidney function during the first year post-AKI, other CKD risk factors, and local resources. Among 96 children with stage 2–3 AKI at a single US center, all children that developed CKD by 3–5 years follow-up had evidence of abnormal eGFR (<90 mL/min/1.73 m^2^) within the first year post-AKI [87]. This suggests that a minimum follow-up period of 1-year may be sufficient to detect most children that will progress to CKD.

In adult AKI survivor clinics, patients are typically followed for approximately one year before discharge or referral to a CKD clinic. In contrast, pediatric AKI survivors are often followed for a longer duration, in some cases up to five years, prior to discharge from AKI survivor clinics [88]. If kidney function remains stable during follow-up in a pediatric AKI clinic, patients should be discharged to their primary care provider (PCP) with a detailed handover summarizing the sentinel event (AKI) (including the timing and duration of the AKI episode, AKI stage, suspected etiology, and details of acute management, such as the use of intravenous fluids, diuretics, and the need for KRT), subsequent clinical course during pediatric AKI clinic follow up, along with recommendations for annual blood pressure monitoring and first-morning urine assessment for proteinuria.

For patients with a stable course for at least one year post-AKI who require transition to adult care due to age, care may similarly be transferred to the PCP. However, in the presence of ongoing concerns, such as unresolved AKI, AKD, CKD, persistent proteinuria, or hypertension, transition to an adult nephrology clinic is warranted, tailored to the specific clinical context. In all transitions, whether to primary care or adult nephrology, the pediatric nephrologist should provide detailed documentation of the aforementioned.

Alnafisi et al. provided a detailed description of the follow-up timeline for pediatric AKI survivors managed at the Hospital for Sick Children, outlining care during the first year post-AKI (Figure 2) as well as in subsequent years. The frequency, duration, and provider of follow-up beyond the first year, similar to considerations during the initial year, depend on AKI severity, the requirement for KRT, and additional risk factors. For example, patients who survived stage 2–3 AKI requiring KRT are followed by a specialist for up to five years, with variable visit frequency, followed by annual monitoring by a primary care provider [86].

### Providers and components of post-AKI surveillance

Decisions regarding appropriate follow-up care provider depends on the perceived risk of CKD and hypertension, based on AKI history and CKD risk factors, and resource availability in the local healthcare system. Children with severe AKI (stage 2–3 or acute kidney replacement therapy), unresolved AKI at discharge, AKD, or preexisting CKD may benefit from referral to a pediatric nephrologist or specialized post-AKI follow-up clinic [81]. Children with stage 1 AKI that experience complete functional recovery should also undergo post-AKI surveillance, but this could be led by their pediatrician, primary care provider, or other specialists involved in their ongoing care. Post-AKI surveillance should include assessment of kidney health (including review of AKI history, blood pressure measurement, eGFR measurement [by serum creatinine or cystatin C], and proteinuria measurement [by urinalysis ± urine protein:creatinine or albumin:creatinine ratio]), patient and family education about AKI and strategies to promote long-term kidney health (e.g., avoiding dehydration and sick-day management), medication review, and counseling about nephrotoxin avoidance [21,81]. Additional laboratory investigations, kidney ultrasound, or other testing (e.g., ambulatory blood pressure monitoring) should be considered on a case-by-case basis.

Established frameworks such as the ‘ABCD’ kidney health assessment (A: AKI history, B: blood pressure measurement, C: creatinine and estimated GFR, and D: drugs list review and determine proteinuria) and the ‘4 M’s’ kidney health response (medication adjustment, minimization of high-risk exposures, messaging to patients and providers, and ongoing monitoring), endorsed by the Canadian Pediatric Society, offer systematic approaches to post-AKI care and education of patients, families, and healthcare providers [81]. These approaches provide a pragmatic framework for post-AKI surveillance among high-risk children until more robust evidence is available to inform optimal follow-up strategies. Electronic health record-based alerts and structured transition-of-care programs may support adherence to follow-up recommendations and ensure early detection of new or progressive CKD [49].

### Neonatal AKI follow-up

Compared to children and adolescents, follow-up guidelines for neonatal AKI are more clearly established. In 2024, the Neonatal Kidney Health Consensus Workshop developed follow-up recommendations for kidney health surveillance among neonatal ICU survivors [89]. Critically-ill infants with a history of stage 2–3 AKI (including acute kidney replacement therapy use), recurrent AKI, or AKI with severe non-renal comorbidity were identified as high-risk individuals. Other high-risk groups were extremely preterm neonates (<28 weeks gestational age), very low birthweight neonates (<1500 grams), and infants with critical cardiac disease. Kidney health evaluation prior to discharge and at 2-years of age was recommended for all high-risk neonates. For critically-ill infants with AKI, kidney health assessment within 6-months of discharge was also recommended and can be used to guide subsequent follow-up.

## Care models and systems-level approaches to post-AKI care

Post-AKI care should commence in the inpatient setting during the critical period of kidney functional recovery, focused on optimizing renal perfusion, managing fluid overload, correcting electrolyte and acid-base disturbances, minimizing ongoing exposure to nephrotoxic medications and radiocontrast, and tailoring medication dosing to estimated GFR [21,88]. In the outpatient setting, dedicated AKI follow-up clinics provide a structured framework for post-discharge surveillance, support by multidisciplinary care, and educate families and healthcare providers about the long-term risks associated with AKI [88]. These clinics facilitate consistent documentation of AKI episodes in the medical record and promote standardized care pathways, including risk-based follow-up schedules and clear criteria for discharge or transition to pediatrician or primary care. Common discharge criteria include stable kidney function, absence of recurrent AKI, and appropriate management of comorbid conditions.

System-level strategies are equally important for improving outcomes across diverse healthcare settings. Electronic health record alerts or registries can help identify children with severe AKI and prompt timely referral for post-AKI surveillance. Education of healthcare providers on AKI, AKD, and nephrotoxin stewardship is critical, particularly for pediatric populations frequently exposed to high-risk medications or states (e.g., cardiac disease, cancer, stem cell or solid organ transplant recipients, diabetes, and sickle cell disease). On a global level, implementation of practical tools is essential, especially in resource-limited settings. The International Society of Nephrology AKI Toolkit provides accessible, evidence-based guidance to support early recognition, management, and follow-up of AKI across a wide range of healthcare environments [90]. By offering standardized care pathways, educational resources, and context-adaptable recommendations, the toolkit helps bridge gaps in post-AKI care worldwide.

## Conclusions

Given the high incidence of AKI in hospitalized children, its established association with long-term renal, cardiovascular morbidity, and neurocognitive impairment, and the lack of standardized pediatric follow-up strategies, enhanced post-AKI surveillance, based on risk stratification, represents a critical opportunity to prevent and detect long-term adverse outcomes. Currently, the absence of reliable biomarkers for identifying AKI survivors at risk of progression to CKD, together with the incomplete integration of AI-based tools into routine clinical practice, underscores the continued importance of structured-longitudinal clinical follow-up post AKI.

## Data Availability

Data sharing is not applicable to this article as no new data were created or analyzed in this study.
